# Stages of change: Strategies to promote use of a Pediatric Early Warning System in resource‐limited pediatric oncology centers

**DOI:** 10.1002/cam4.6087

**Published:** 2023-07-05

**Authors:** Marisa Cristin Woo, Gia Ferrara, Maria Puerto‐Torres, Srinithya R. Gillipelli, Paul Elish, Hilmarie Muniz‐Talavera, Alejandra Gonzalez‐Ruiz, Miriam Armenta, Camila Barra, Rosdali Diaz‐Coronado, Cinthia Hernandez, Susana Juarez, José de Jesús Loeza, Alejandra Mendez, Erika Montalvo, Eulalia Peñafiel, Estuardo Pineda, Dylan E. Graetz, Teresa Kortz, Asya Agulnik

**Affiliations:** ^1^ University of California San Francisco California USA; ^2^ St. Jude Children's Research Hospital Memphis Tennessee USA; ^3^ Baylor College of Medicine Houston Texas USA; ^4^ Rollins School of Public Health Emory University Atlanta Georgia USA; ^5^ Abt Associates Rockville Maryland USA; ^6^ Hospital General de Tijuana Tijuana Mexico; ^7^ Hospital Dr. Luis Calvo Mackenna Santiago Chile; ^8^ Instituto Nacional de Enfermedades Neoplásicas Lima Peru; ^9^ Hospital Infantil Teletón de Oncología Querétaro Mexico; ^10^ Hospital Central Dr. Ignacio Morones Prieto San Luis Potosí Mexico; ^11^ Centro Estatal de Cancerología Xalapa Mexico; ^12^ Hospital Oncológico SOLCA Núcleo de Quito Quito Ecuador; ^13^ Instituto del Cáncer SOLCA Cuenca Cuenca Ecuador; ^14^ Hospital Nacional de Niños Benjamín Bloom San Salvador El Salvador

**Keywords:** behavioral science, clinical cancer research, clinical management, implementation science, pediatric cancer, Pediatric Early Warning Systems, resource‐limited, stages of change, transtheoretical model

## Abstract

**Background:**

Pediatric Early Warning Systems (PEWS) assist early detection of clinical deterioration in hospitalized children with cancer. Relevant to successful PEWS implementation, the “stages of change” model characterizes stakeholder support for PEWS based on willingness and effort to adopt the new practice.

**Methods:**

At five resource‐limited pediatric oncology centers in Latin America, semi‐structured interviews were conducted with 71 hospital staff involved in PEWS implementation. Purposive sampling was used to select centers requiring variable time to complete PEWS implementation, with low‐barrier centers (3–4 months) and high‐barrier centers (10–11 months). Interviews were conducted in Spanish, professionally transcribed, and translated into English. Thematic content analysis explored “stage of change” with constant comparative analysis across stakeholder types and study sites.

**Results:**

Participants identified six interventions (training, incentives, participation, evidence, persuasion, and modeling) and two policies (environmental planning and mandates) as effective strategies used by implementation leaders to promote stakeholder progression through stages of change. Key approaches involved presentation of evidence demonstrating PEWS effectiveness, persuasion and incentives addressing specific stakeholder interests, enthusiastic individuals serving as models for others, and policies enforced by hospital directors facilitating habitual PEWS use. Effective engagement targeted hospital directors during early implementation phases to provide programmatic legitimacy for clinical staff.

**Conclusion:**

This study identifies strategies to promote adoption and maintained use of PEWS, highlighting the importance of tailoring implementation strategies to the motivations of each stakeholder type. These findings can guide efforts to implement PEWS and other evidence‐based practices that improve childhood cancer outcomes in resource‐limited hospitals.

## Graphical abstract

This multicenter, multinational qualitative study identifies strategies used by implementation leaders to promote hospital staff's decision to adopt a Pediatric Early Warning System (PEWS) in resource‐limited pediatric oncology centers. These findings can guide efforts to implement PEWS and other quality improvement tools that improve childhood cancer outcomes globally.

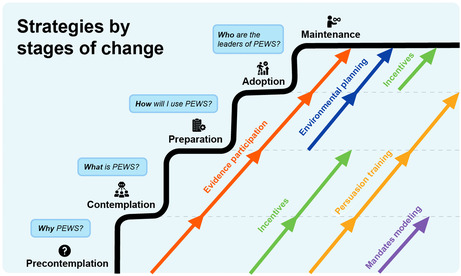

## 1 BACKGROUND

Approximately 40% of pediatric oncology patients require critical care during their cancer treatment.^1^ Pediatric oncology patients admitted to the pediatric intensive care unit (PICU) experience a higher rate of mortality compared to the general PICU population, particularly in resource‐limited hospitals.^1^, ^2^ Pediatric Early Warning Systems (PEWS) are quality improvement tools used to facilitate early detection of critical illness.^3^ PEWS consist of a scoring tool and action algorithm to identify and monitor hospitalized children at risk of clinical deterioration.^4^ Bedside nurses evaluate vital signs, behavioral indicators, and relative concern to calculate a standardized score.^4^ This score is associated with an action algorithm that guides the clinical team's response.^4^ PEWS have been shown to reduce clinical deterioration events and PICU utilization, improve interdisciplinary and provider‐family communication, enhance perceptions of healthcare quality, support clinician emotions, and result in cost‐savings.^4^, ^5^, ^6^, ^7^, ^8^, ^9^, ^10^ However, PEWS are rarely used in resources‐limited hospitals, in part due to challenges during implementation.^3^

Prior work identified stakeholder “stage of change” as integral to successful PEWS implementation.^11^ Stakeholders are individuals with interest and influence to affect implementation of evidence‐based practices like PEWS.^12^ The stages of change are phases of readiness that describe stakeholder willingness to adopt and use a new practice: precontemplation (resistant to behavior change), contemplation (ambivalent towards behavior change), preparation (expressing interest in a plan of action to change behavior), adoption (changing behavior), and maintenance (independently sustaining behavior change).^13^

The Capability, Opportunity, Motivation—Behavior (COM‐B) model is a behavior change model that explains how stakeholders progress through the stages of change.^14^ By explaining what a stakeholder needs for their perceptions towards PEWS to change, this model can guide implementation planning to tailor strategies to unmet stakeholder needs and promote adoption of evidence‐based practices like PEWS. This study uses behavior change theory and the COM‐B model to explore strategies used by implementation leaders to successfully promote stakeholder adoption and maintained use of PEWS in resource‐limited pediatric oncology centers.

## 2 METHODS

We conducted a secondary analysis of PEWS implementation in resource‐limited pediatric oncology centers.^11^ The primary analysis identified barriers and enablers in the domains of characteristics of individuals (clinical staff), inner (hospital) setting, outer setting (external factors), the PEWS intervention, and implementation process.^11^ The stages of change were a major theme identified in the inner setting domain as an important barrier or enabler of implementation success.^11^ This secondary analysis evaluated the process by which implementation leaders successfully converted implementation barriers into enablers for clinical staff using a stages of change framework. We followed Consolidated Criteria for Reporting Qualitative Research (COREQ) guidelines.^15^ Methodology for the primary study was previously described^11^ and is summarized below.

### 2.1 Site and participant sampling

Escala de Valoración de Alerta Temprana (EVAT) is a Spanish‐language PEWS validated in pediatric oncology patients.^16^ Proyecto EVAT is a multicenter quality improvement collaborative to scale up PEWS in Latin America.^11^ We recruited Proyecto EVAT centers who completed PEWS implementation prior to March 2020. From 23 centers meeting these criteria, we used purposive sampling to select low‐barrier (requiring 3–4 months from pilot initiation to successful implementation) and high‐barrier sites (10–11 months). A local study lead identified 10–15 clinical and administrative hospital staff involved in PEWS implementation at each center to participate in a semi‐structured interview (estimated number required for thematic saturation).^17^ Research team members then contacted identified staff for recruitment to the study; all identified participants agreed to participate and completed interviews.

### 2.2 Data collection

An interview guide assessing barriers and enablers to PEWS implementation (Figure S1) was developed in English, translated into Spanish, and iteratively revised.^18^ We piloted the interview guide with three individuals from a Proyecto EVAT center not recruited for this study. From June to August 2020, bilingual interviewers (PE, SG) conducted virtual semi‐structured interviews using WebEx. These interviewers did not have a prior relationship with participants, did not work at the centers, and were not involved in Proyecto EVAT. Interview duration was approximately 1 h. Bilingual interviewers (PE, SG) conducted interviews in Spanish and audio recorded. A professional translation company transcribed, deidentified, and translated the interviews into English for analysis.

### 2.3 Analysis

Two analysts (AA, GF) created a codebook (Table S1) with codes developed a priori and inductively based on iterative review of nine transcripts.^18^ The analysts then independently coded all transcripts using MAXQDA software, achieving a kappa of 0.8–0.9, with discrepancies resolved by a third analyst (DG).

For this study, thematic content analysis was used to explore segments coded as “stage of change”, defined as “willingness, or lack of willingness of individuals or authorities in the hospital to gain new skills, accept change, or show interest/enthusiasm for PEWS” (Table S1). Relevant to successful implementation, the stages of change describe a stakeholder's evolving perception towards PEWS. We chose the COM‐B model for its description of factors affecting an individual's willingness to change their behavior and move through the stages of change: physical and psychological capability to understand how to do the behavior, physical and social opportunity to provide the necessary resources, and reflective and automatic motivation to create inner drive.^14^

Through iterative review of transcripts, we identified strategies used by PEWS implementation leaders to promote stakeholder movement through each stage of change and categorized these based on the behavior change wheel, a framework that describes behavior change strategies.^14^ The behavior change wheel defines strategical actions used to address a stakeholder's needs and thus change a stakeholder's perception of PEWS. We used constant comparative analysis to explore how strategies related to stakeholder current stage of change, COM‐B factors, and different stakeholder types.

### 2.4 Ethical considerations

The St. Jude Children's Research Hospital Institutional Review Board approved this study as exempt with minimal risk; study participants provided verbal consent at the beginning of each interview. We obtained additional ethics approval from participating centers as necessary.

## 3 RESULTS

The 71 participants included nurses (45%), physicians (45%), and hospital administrators and data managers (10%) from three low‐barrier centers (San Luis Potosi (SLP), Mexico; Cuenca, Ecuador; San Salvador, El Salvador) and two high‐barrier centers (Xapala, Mexico; Lima, Peru; Tables S2 and S3). Participants identified two stakeholder types important to PEWS adoption: clinical staff responsible for using PEWS in patient care and hospital directors who approved policies supporting hospital‐wide PEWS implementation.

Participants identified six interventions (strategic actions that did not require formal approval)—training, incentives, participation, evidence, persuasion, and modeling—and two policies (directives needing leadership approval)—environmental planning and mandates—that promoted stakeholder adoption and maintained use of PEWS. Implementation leaders used different strategies to address each stage of change and COM‐B factor.

### 3.1 Strategies across stages of change

**FIGURE 1:**
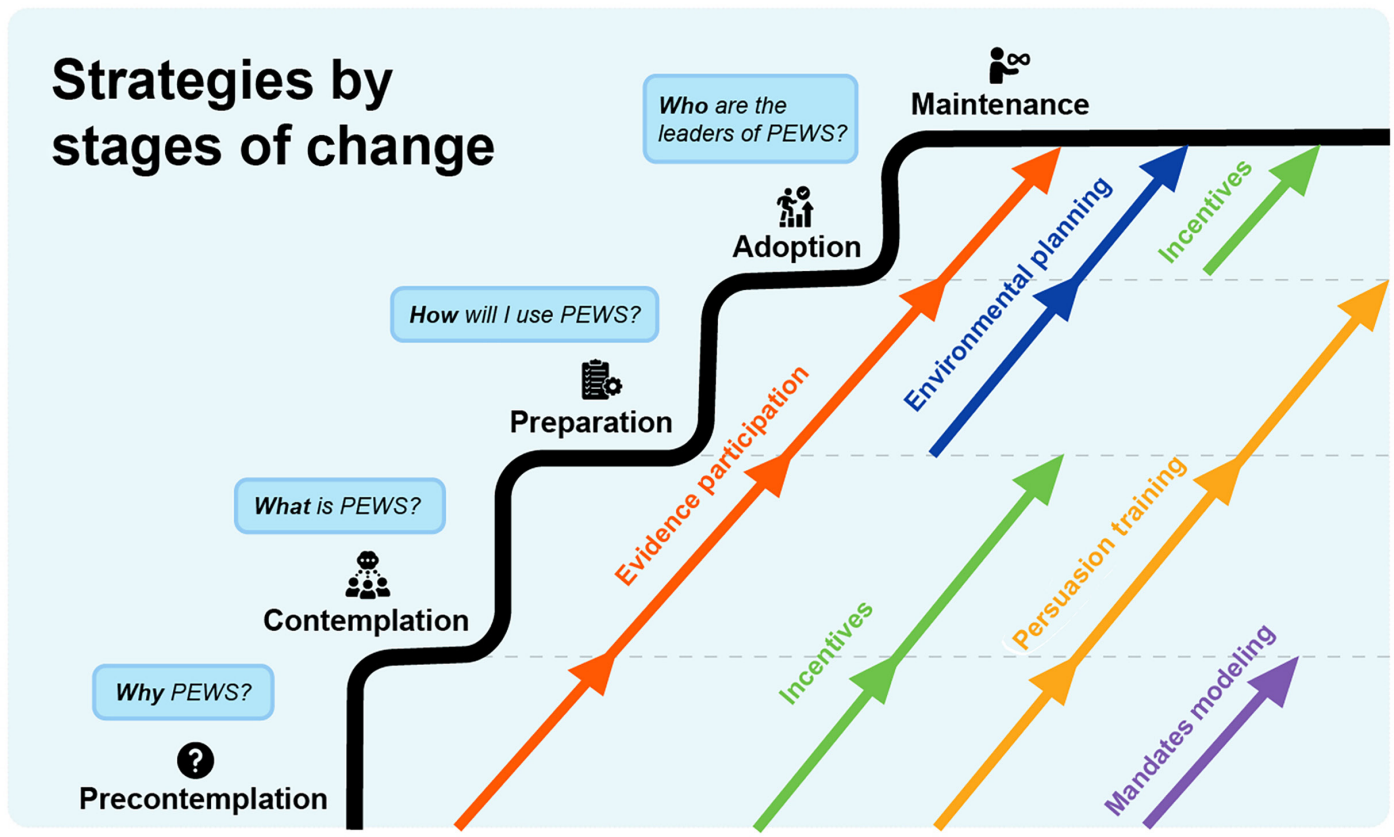
Identified strategies mapped to stage of change. This figure describes the strategies that implementation leaders used to promote stakeholders' movement through the stages of precontemplation, contemplation, preparation, adoption, and maintenance. Stakeholders had distinct concerns in each stage. To move stakeholders from one stage to the next, implementation leaders used different collections of strategies that addressed concerns unique to each stage. Some strategies answered questions about the use of PEWS for stakeholders in every stage, while other strategies addressed stakeholder concerns relevant to only a few stages. PEWS, Pediatric Early Warning System.

Stakeholders presented in various stages of change throughout the implementation process (Table 1). Implementation leaders, therefore, employed distinctive strategies to address different concerns associated with each stage of change (Table 2, Figure 1).

**TABLE 1:** Stakeholders in each stage of change.

Stage of change	Transtheoretical model^13^	Identified themes	Example
Precontemplation	People present as resistant and do not intend to change in the foreseeable future.	Stakeholders are resistant towards using PEWS.	“At the beginning, some nurses even denied doing [PEWS]. I mean they would fill in the whole nursing sheet except the added part [for PEWS]” (implementation leader, SLP)
Contemplation	People present as ambivalent and intend to change in the next 6 months.	Stakeholders are ambivalent towards using PEWS.	“We always think, here comes a new program. We need to work more, organize, support. Sometimes there are programs with very short goals and at the first moment, there's some kind of apathy. There's a new program, we'll see” (nurse director, Xapala)
Preparation	People have a plan of action and intend to change in the immediate future.	Stakeholders demonstrate interest in using PEWS.	“The more we knew about PEWS, the more interested we felt about the project. And we said, yes, we can apply PEWS” (implementation leader, Cuenca)
Adoption	People have made specific behavioral modifications within the past 6 months.	Stakeholders take action to use PEWS or support policies to implement PEWS.	“The hospital has accepted PEWS as part of the staff's work. So, [hospital directors] give us the sheets. They open the doors for the training. They include us in their training session of their projects and programs, even in the medical part, with the residents, the new nurses” (implementation leader, Lima)
Maintenance	People do not use change processes as often and become increasingly confident in maintaining their behavior change.	Stakeholders independently use PEWS and actively promote the use of PEWS.	“I think it's excellent. It's a scale that is applied to all oncology patients. We do not need to insist or remember. No, it's something that is done in a natural way, all the staff” (quality director, San Salvador)

Abbreviations: PEWS, Pediatric Early Warning System; SLP, San Luis Potosi.

**TABLE 2:** Identified strategies mapped to stage of change.

Stage of change	Strategy to promote movement to the next stage of change	Example
Precontemplation	Training	“[Nurses] used to say no, I have work to do. I do not have time; but with training, they realized their work was going to be better, it was going to be optimal” (human resource administrator, Cuenca)
	Incentives	“[Nurses] would say no, that [training] time must be paid. So, we would arrange the training sessions on working hours in order to do it” (implementation leader, San Salvador)
	Participation	“[Some nurses] have kind of resistance to technology… Include them in the construction of the program from the beginning so they do not feel it like an imposition but as part of the nature of the job they are doing” (foundation administrator, San Salvador)
	Evidence	“ICU doctors were not too open about [PEWS] at the beginning, but as they are seeing results, they are getting more involved” (nurse director, Lima)
	Persuasion	“[The pilot] made us understand that [PEWS] was nothing more than systematizing information that we were already taking from the patients. [This] was something important for the nurses to realize that it was not to increase the workload but simply to organize what we were doing” (physician director, Lima)
	Modeling	“I think [Physician] helped a lot because she convinced the residents that this was something good and from there, we saw a change in [residents'] attitude and willingness to use the scale” (implementation leader, SLP)
	Mandates	“We had a few nurses reluctant to do the rating and use the scale. So, obviously that changed with the intervention of the [nursing director]. There was some movement in the staff” (implementation leader, Lima)
Contemplation	Training	“We take vital signs one time during the day and two times during the night. So, make the staff understand that they need to take vital signs more frequently for children depending on their category in the scale” (implementation leader, San Salvador)
	Incentives	“To get more people to train… I think it's always important the thinking of they are offering me a course, I'm going somewhere else, I will meet new colleagues, and that generates interest” (implementation leader, Xapala)
	Participation	“[Pediatric staff] all know the project, they all participate in the project, all of them has developed the project. So I think that was a great strategy, not to include just a few people, but the entire department of pediatrics” (research director, Cuenca)
	Evidence	“We showed [hospital directors] the results [PEWS] had in other places. So, I think [hospital directors] got convinced in that moment, and they supported us… We wanted to replicate it in the hospital” (implementation leader, Xapala)
	Persuasion	“So, when [implementation leaders] proposed [PEWS], the hospital in general agreed. Low costs… a better life for the patients” (implementation leader, Cuenca)
Preparation	Training	“It's very important that we know [PEWS] because when they deliver the patient transferred from another service, they say we transfer him here because of this diagnosis and also, he's got a PEWS scale of this punctuation. At the moment they mention the punctuation, we already know that he's a patient that started to deteriorate and requires a more specialized attention to prevent the worst situation” (nurse director, SLP)
	Participation	“In general, the staff understood what PEWS was and tried to adapt to the format… We did make modifications and based on that pilot, the implementation was easier” (quality director, Xapala)
	Evidence	“We have patients that stay only a few days at the hospital, less patients at ICU, etc. So, [clinical staff] see results, they see less work for them” (data manager, Xapala)
	Persuasion	“[Nurses] just had to organize what they were doing, and that's what they were told at the training. You do this already but now you are going to organize it and register it, nothing else. That's what made it easier” (data manager, Xapala)
	Environmental planning	“We keep a sheet for vital functions, and the nurses would fill in [that] sheet and the PEWS form. So, we talked to the hospital to merge the two sheets to not work double… This made the staff feel better” (implementation leader, Lima)
Adoption	Incentives	“At the end, PEWS became a pride for the institution because we are [recognized as] a center of excellence… This has a repercussion in people wanting to be part of the project” (physician director, Lima)
	Participation	“I think now [nurses] come voluntarily, with better mood, because they feel they are part of the results and the progress. It's not like [nurses] used to do it before [when] they felt an obligation to assist to the classes… Now, I see they are motivated” (implementation leader, Cuenca)
	Evidence	“After the results, [hospital directors] say it's a good project that has to continue and keep its sustainability” (nurse director, Cuenca)
	Environmental planning	“[PEWS] works wonderfully. If you want to know the condition of a patient, you just go to the [PEWS] database and you can see all the ratings given by the nurse” (data director, San Salvador)

Abbreviations: ICU, intensive care unit; PEWS, Pediatric Early Warning System; SLP, San Luis Potosi.

To move stakeholders from the precontemplation to contemplation stage, implementation leaders explained why they should use PEWS: “*We knew* [*PEWS*] *was going to generate certain rejection*… *At the end*, [*physicians*] *finally understood that by having* [*the patient*] *more controlled and monitored*, *I can transfer him calmly*, *using a more secure method*, *and intubate him where I have a ventilator and all the equipment*” (implementation leader, Xapala). These strategies motivated stakeholders to learn about PEWS benefits, with additional strategies needed to move stakeholders beyond contemplation: “*Even though there were people unconvinced*, *the hospital's directors gave the order to start with this*, *and* [*clinical staff*] *had to do it*. *But as time passed*, *I think* [*clinical staff*] *convinced themselves*” (implementation leader, SLP).

Implementation leaders showed examples of PEWS' feasibility at other, similar hospitals to move stakeholders from contemplation to preparation: “*I learned a little bit about the experience in the Dominican Republic*, *Honduras*… *how had they tropicalized PEWS for them* [*for the Caribbean and Latin American tropics*]. *That was a point of reference for us to take it to our hospital*” (nurse director, San Salvador).

Implementation leaders demonstrated PEWS' feasibility specifically in the local hospital setting, and as stakeholders learned how to use PEWS, stakeholders moved from preparation to adoption: “*The pilot showed that PEWS worked*, *and this maybe opened the doors to create the conditions to run it as a quality project from the hospital*” (physician director, San Salvador). Additional environmental planning provided physical resources necessary to use PEWS: “*When we had all the material*, *the boards updated*… *the first thing* [*physicians*] *do is check the sheet and see if someone has a red or yellow* [*elevated*] *PEWS*, *so they can start to work on that patient*” (nurse director, Lima).

To move stakeholders from adoption to maintenance, implementation leaders encouraged stakeholders to feel pride as leaders in the program: “*When* [*clinical staff*] *talk about PEWS*, *you can see their enthusiasm about the project*. *They own the project*, *and I think that was essential*. *Because the director can be excited*, *but if the people in charge of applying it are not involved and convinced*, *it would be more difficult*” (nurse director, San Salvador).

### 3.2 Strategies across COM‐B factors

Participants from all centers described a need to holistically address all factors affecting stakeholders' decision to adopt PEWS: “*We realized that in some people*, *the training gave knowledge*, *but there was no attitude to do it*… *They had the knowledge*; *they knew* [*PEWS*] *was good*, *but they simply didn't have the desire to do it*” (implementation leader, Lima). Collectively, the identified strategies addressed every COM‐B factor by providing the knowledge (capability), resources (opportunity), and desire (motivation) to use PEWS (Table 3).

**TABLE 3:** Identified strategies mapped to addressed COM‐B factors^a^.

COM‐B behavioral factors	COM‐B definition^14^	Training	Incentives	Participation	Evidence	Persuasion	Modeling	Environmental planning	Mandates
Physical capability	People have developed the skills and abilities necessary to change their behavior.	X							
Psychological capability	People have the knowledge necessary to regulate their behavior.	X			X				
Physical opportunity	People have the environmental context and resources necessary to change their behavior.							X	
Social opportunity	People have social influences from social pressure, norms, and social comparisons.			X			X		X
Automatic motivation	People have inner drive derived from emotions, rewards, and punishment.		X			X		X	X
Reflective motivation	People have beliefs about their capabilities, behavioral consequences, professional roles, goals, and intentions.		X	X	X	X			X

Abbreviation: COM‐B, Capability, Opportunity, Motivation—Behavior.

^a^ Adapted from Michie S, van Stralen MM, West R.^14^

#### 3.2.1 Training

Implementation leaders from all centers provided clinical staff with relevant knowledge and skills needed to use PEWS: “*It was useful* [*that hospital staff*] *all attended the first training*. *That was the way to let them know what PEWS was*, *and that made them want to participate in the project*” (implementation leader, Cuenca). Clinical staff practiced using PEWS during training sessions and developed further confidence: “*When* [*nurses*] *took the* [*PEWS*] *sheet into practice*, *they started to realize how easy and feasible* [*it*] *was to apply it*; *but I think at the beginning*, *as in every project*, *the fear of the unknown is what created more resistance*” (nurse director, San Salvador).

#### 3.2.2 Incentives

At all centers, implementation leaders motivated clinical staff with recognition and praise for exceptional effort in consistently and accurately applying PEWS. One low‐barrier center provided academic credit to recognize participation in PEWS training: “[*Training certificates*] *were* [*accredited*] *by the university*. *Nurses and residents didn't feel* [*training*] *as a burden to assist*. *At the same time*, *they had a guaranteed academic reward that they could use in their curriculum* [*vitae*]” (implementation leader, Cuenca). Incentives associated with PEWS use, like evaluation scores, created immediate motivation: “*From the moment* [*clinical staff*] *start working here*, *they start with training and evaluations*. *I think a training gives results*, *and* [*clinical staff*] *are more motivated with the training when they know an evaluation is coming*” (implementation leader, Cuenca).

#### 3.2.3 Participation

Implementation leaders from all centers encouraged staff participation, including providing feedback on PEWS. This empowered stakeholders to directly contribute to improving the program: “*It's been a participation process with the colleagues regarding the modifications we've done so the* [*PEWS*] *sheet and the interpretation of* [*PEWS*] *wouldn't be too difficult*” (implementation leader, San Salvador). Participants from all centers emphasized the need to present PEWS as a multidisciplinary, rather than a personal, project: “*This is so important that* [*PEWS*] *is not just a thesis' interest*… *It's easier if I'm going to do work that will improve the attention to patients* [*rather*] *than taking vital signs*, *doing an evaluation because someone wants to publish a paper*” (physician director, SLP).

#### 3.2.4 Evidence

Implementation leaders frequently shared data and clinical observations that demonstrated PEWS effectiveness. This evidence increased knowledge and influenced stakeholders' perceptions about PEWS' utility, motivating them to continue using PEWS to improve patient care: “*To see that the children* [*went*] *to the ICU earlier*… *so a child has less complications*. *That is an important reinforcement for all of us*, *because there we can see the value of the scale*” (physician director, Cuenca).

#### 3.2.5 Persuasion

Implementation leaders from all centers emphasized PEWS benefits directly related to stakeholders' individual interests: “[*We are*] *looking for a way to save money for the hospital*. *Actually*, *we sold the idea from that argument*… *The savings we make* [*by*] *preventing a child from going to the ICU*, *and with that money*, [*we can*] *buy treatment for three children*” (implementation leader, San Salvador).

#### 3.2.6 Modeling

Implementation leaders and “champions”, peers who enthusiastically supported PEWS, served as role‐models: “[*A nurse*] *got very involved*, *she was a regular nurse just like the rest*… *They saw this person that played a key role for the collaboration of all nurses*” (physician director, Cuenca). Champions with a similar professional background modeled PEWS' feasibility: “*We did have reluctance at the beginning*… *But after we started* [*PEWS*] *only on the morning shift*, [*the other shifts*] *got interested and finally we now apply* [*PEWS*] *on all shifts*” (nurse director, SLP).

#### 3.2.7 Environmental planning

Hospital policies structurally integrated PEWS into the physical work environment. For example, all centers created a PEWS display board that centralized patient clinical data: “[*The board*] *is a very useful tool because I didn't have to visit each of the patients*. *I would go to the board and knew the clinical state of every patient or who required more attention*” (implementation leader, Xapala).

#### 3.2.8 Mandates

Hospital directors, such as the medical director or nursing chief, set institutional norms and required clinical staff to use PEWS. Hospital directors enforced mandates and motivated clinical staff to avoid reprimands: “*We had some red patients* [*with high PEWS scores*] *that even died in the service*… *In those analysis*, *the chiefs of divisions are involved*. *They are the bosses of those residents*. *Well*, *they started to scold them*, *and this is why* [*residents*] *are now interested*” (nurse director, SLP).

### 3.3 Strategies across stakeholder types

Table 4 describes how the different responsibilities and roles of clinical staff and hospital directors required implementation leaders to tailor specific strategies to stakeholder type, depending on each individual's specific role in PEWS. Some strategies addressed the interests of both clinical staff and hospital directors, while other strategies targeted interests specific to the role of clinical staff.

**TABLE 4:** Identified strategies mapped to stakeholder type.

Strategy type	Strategy	Stakeholder type	Stakeholder‐specific interests addressed by each strategy	Example
Intervention	Training	Clinical staff	Skills and knowledge to use PEWS in clinical practice	“We also had to train these residents… to show them what PEWS is and how important it is to respond [to] nursing warnings” (implementation leader, Lima)
	Incentives	Clinical staff	Positive emotions associated with praise and recognition, academic credit for career advancement	“We would give a certificate of recognition to the people who would have less errors [with PEWS] or [to the] people with a lot of mistakes but in the next month got better” (implementation leader, Lima)
	Participation	Clinical staff	Responsive work culture, perceived agency to contribute to improved patient care	“We used to meet constantly to find out what we were missing, the feedback they gave us. We even formed an investigation team among nurses where they measured the satisfaction among the nursing staff” (implementation leader, Lima)
	Evidence	Clinical staff	Improvement of patient care	“It's a very good tool. It has helped to detect patients on time, and it has given us the satisfaction that those patients got better and are in great conditions” (implementation leader, Lima)
		Hospital directors		“The data they presented [to] us: indicators, the impact of mortality decrease, also the impact in the survival of a patient with cancer… [It] allowed us to decide” (physician director, Lima)
	Persuasion	Clinical staff	Reduced workload, loving emotions related to the care of children	“I had to convince nurses who are mothers first, to make them understand that the child should be treated as if it was their own child” (nurse director, Lima)
		Hospital directors	Benefit to the hospital's image, reduced financial costs	“When we heard St. Jude [was participating], this is a world reference in the treatment of cancer in children. Since then, we knew we could participate in that project” (research director, Cuenca)
	Modeling	Clinical staff	Demonstrated feasibility to adopt PEWS	“We even have people… that accepted it so well. They applied it and adapted to it so fast, that even they were the ones motivating their own colleagues, saying look this helps in this way” (nurse director, Xapala)
		Hospital directors		“After seeing the enthusiasm the PEWS team had, the only thing left to do was to support them to make it possible” (quality improvement director, San Salvador)
Policy	Environmental Planning	Clinical staff	Reduced workload, ease of PEWS use in physical work environment	“At the beginning, [PEWS] was seen as extra work, more papers… On the contrary, [PEWS] is less work because… [The nursing note] explained in one page was going to have all the information simplified” (implementation leader, San Salvador)
	Mandates	Clinical staff	Avoidance of reprimands from superiors, adherence to professional hierarchy	“Even though there were [clinical staff] that were not convinced, the hospital's direction gave the order to start with this, and they had to do it” (implementation leader, SLP)
		Hospital directors		“When the [nurse director] accepted the project, it was simpler because she told the rest of the chiefs from all areas of hospitalization that PEWS is going to be applied. That was it, and this was a part of the job” (implementation leader, Lima)

Abbreviation: PEWS, Pediatric Early Warning System; SLP, San L.uis Potosi.

Evidence, persuasion, modeling, and mandates targeted both clinical staff and hospital directors. These strategies demonstrated institutional feasibility of PEWS: “*We got motivated when we heard* [*PEWS*] *works in Haiti and Cuba*. *So*, *we thought if they could*, *why wouldn't we?*” (implementation leader, San Salvador).

Clinical staff focused on the impact of PEWS on their daily work: “[*A patient*] *had* [*an elevated*] *yellow PEWS* [*score*]. *He didn't require ICU attention*, *but everything was so well done that the patient recovered*. *There*, [*clinical staff*] *realized that* [*PEWS*] *is important*. [*PEWS*] *will improve the attention for the patient and will also help them in their job because they will no longer have a patient who is in critical condition*” (implementation leader, Lima). Training, incentives, participation, and environmental planning specifically targeted clinical staff. PEWS implementation required clinical staff training to develop skills and knowledge, and environmental planning to provide physical resources to use PEWS: “[*PEWS*] *is applied by the nurses*. *They had to be trained and learn a new way of taking vital signs*” (implementation leader, Xapala). Incentives and participation recognized and encouraged the active role of clinical staff in PEWS: “*I think the positive reinforcement that nurses get from the nurse leaders* [*is*] *that the work is well done*, *that they have a good attitude*. *That we are a team*, *and we must stay together for the children*, *like the pediatrician with the residents*. *That big motivation* [*is*] *to listen to the nurses*” (physician director, Cuenca).

### 3.4 Strategies across high‐ and low‐barrier centers

High‐ and low‐barrier centers differed in the amount of time required to move from pilot initiation to successful implementation. Implementation leaders at high‐ and low‐barrier centers differed in their application of some strategies, impacting time required for implementation.

While all low‐barrier centers and one high‐barrier center used incentives, one high‐barrier center did not describe the use of any incentives. Without use of incentives, one high‐barrier center waited on outcomes data to motivate staff, thus delaying implementation: “*You'll have to spend some time to do measurements*… *I think in this case it took the doctor 18 months or 24 months*. [*The doctor*] *presents the results*, *and that's when everybody is convinced with the importance of the implementation*” (quality director, SLP).

Similarly, PEWS adoption by hospital directors allowed for institutional policies, like mandates, that facilitated dissemination of PEWS: “*Our directors and the people chosen to implement the project have credibility*, *leadership*… *without those*, *nobody would have paid attention*” (data director, San Salvador). Implementation leaders from low‐barrier centers involved hospital directors during the pre‐implementation planning phase. In contrast, high‐barrier centers waited longer to involve hospital leaders, delaying implementation: “*The* [*nurse director*] *would put barriers*. *If* [*the nurse director*] *was doing that*, *the rest of the nurses would never feel like* [*PEWS*] *was something that they should do*” (physician director, SLP). Early engagement of hospital directors in the PEWS implementation process allowed for earlier use of these effective strategies, thus reducing barriers to implementation.

## 4 DISCUSSION

This study identified six interventions and two policies used by implementation leaders to successfully guide clinical staff and hospital directors through the stages of change to promote adoption and maintained use of PEWS in resource‐limited pediatric oncology centers. Collectively, the identified strategies addressed all six COM‐B factors required for behavior change, supporting the relevance of the COM‐B model in these settings. High‐ and low‐barrier centers used strategies differently, offering potential explanation for different length of time required for implementation. Recognizing different stakeholders' priorities and readiness to accept a new practice, implementation leaders tailored strategies to each stakeholder's stage of change, unmet needs, and specific interests.

Stakeholder stage of change, influencing the decision to adopt and continue using a new behavior, is important for successful implementation of any evidence‐based practice.^13^ In previous work, an important determinant of sustainability and ongoing use of PEWS was hospital staff's perceptions of PEWS' relative importance and impact in patient care.^10^, ^19^, ^20^ By using a stages of change framework to understand how stakeholders' perceptions towards PEWS change, this analysis provides further insight into how stakeholders choose to adopt new clinical practices, and how adoption can be promoted through targeted strategies. Identified differences between strategies used at low‐ and high‐barrier centers suggests timing of stakeholder engagement influences implementation time, an outcome particularly relevant to resource‐limited settings where longer implementation may require more resources and result in premature implementation abandonment.^21^ Based on these findings, we recommend early engagement of hospital directors during planning of any program to implement a new clinical practice. Similarly, incentives and mandates can effectively overcome initial staff resistance to change, promoting early adoption. We also recommend active application of strategies informed by behavior change theory to facilitate implementation in resource‐limited hospitals, where challenges with implementation may perpetuate health disparities by delaying uptake of effective clinical practices.^22^ Implementation strategies that utilize behavior change theory are less frequently applied to plan implementation in these settings.^22^, ^23^ By leveraging behavior change theory to identify strategies for adoption, this study demonstrates how such work can be used to promote equitable implementation of effective clinical practices across variable resource‐levels.

### 4.1 Limitations

This study has several limitations. The study only included centers that completed PEWS implementation as all Proyecto EVAT centers which started eventually successfully implemented PEWS.^24^ Thus, these findings have limited generalizability to other evidence‐based practices that may have poor uptake or more frequent implementation failure. It is likely, however, that other factors, such as public health crises, political unrest, and resource challenges, rather than stakeholder buy‐in, are more relevant to these early implementation challenges.^24^ This study's focus on stakeholder stage of change is most relevant to hospitals actively planning and implementing new interventions. This study also focused on pediatric oncology centers implementing one evidence‐based practice; thus, our findings may not be generalizable to centers implementing interventions for other populations. However, this study integrated multiple behavior‐change theories with empiric evidence about implementation, thus strengthening our findings' validity to understand and contextualize participants' experiences. This study provides insight into strategies to move stakeholders from the precontemplation to maintenance stage for clinical practices in resource‐limited hospitals.

### 4.2 Conclusions

In this multicenter, multinational analysis, we identified strategies used by implementation leaders to promote stakeholder movement from resistance to adoption and enthusiastic, maintained use of PEWS. These findings can guide practitioners and researchers implementing PEWS or other evidence‐based practices in hospitals of variable resource‐levels, ultimately promoting equity in childhood cancer outcomes globally.

## AUTHOR CONTRIBUTIONS

**Marisa Cristin Woo:** Formal analysis (equal); visualization (equal); writing – original draft (equal); writing – review & editing (equal). **Gia Ferrara:** Data curation (equal); formal analysis (equal); writing – review & editing (equal). **Maria Puerto‐Torres:** Data curation (equal); project administration (equal); **Srinithya R. Gillipelli:** Investigation (equal); writing – review & editing (equal). **Paul Elish:** Investigation (equal); writing – review & editing (equal). **Hilmarie Muniz‐Talavera:** Investigation (equal); writing – review & editing (equal). **Alejandra Gonzalez‐Ruiz:** Data curation (equal); project administration (equal); writing – review & editing (equal). **Miriam Armenta:** Investigation (equal); writing – review & editing (equal). **Camila Barra:** Investigation (equal); writing – review & editing (equal). **Rosdali Díaz‐Coronado:** Investigation (equal); writing – review & editing (equal). **Cinthia Hernandez:** Investigation (equal); writing – review & editing (equal). **Susana Juarez:** Investigation (equal); writing – review & editing (equal). **José de Jesús Loeza:** Investigation (equal); writing – review & editing (equal). **Alejandra Mendez:** Investigation (equal); writing – review & editing (equal). **Erika Montalvo:** Investigation (equal); writing – review & editing (equal). **Eulalia Peñafiel:** Investigation (equal); writing – review & editing (equal). **Estuardo Pineda:** Investigation (equal); writing – review & editing (equal). **Dylan E. Graetz:** Conceptualization (equal); methodology (equal); formal analysis (equal); writing – review & editing (equal). **Teresa Kortz:** Formal analysis (equal); supervision (equal); writing – original draft (equal); writing – review & editing (equal). **Asya Agulnik:** Conceptualization (equal); methodology (equal); formal analysis (equal); resources (equal); supervision (equal); writing – original draft (equal); writing – review & editing (equal).

## FUNDING INFORMATION

This study was funded by the American Lebanese‐Syrian Associated Charities (ALSAC). Dr. Agulnik was funded by the Conquer Cancer Foundation Global Oncology Young Investigator Award, and Dr. Kortz was funded by the National Institute of Allergy and Infectious Diseases of the National Institutes of Health for this work. These funders were not involved in the design or conduct of the study; collection, management, analysis, or interpretation of the data; preparation, review, or approval of the manuscript; or decision to submit the manuscript for publication.

## CONFLICT OF INTEREST STATEMENT

The authors declare no competing interests.

## ACCESS TO DATA AND DATA ANALYSIS

Marisa Woo, Asya Agulnik, and Gia Ferrara had full access to all the data in the study and take responsibility for the integrity of the data and the accuracy of the data analysis.

## Supporting information


Data S1.

## Data Availability

The data that support the findings of this study are available from the corresponding author upon reasonable request.
